# Non-pharmacological interventions for postpartum depression: A protocol for systematic review and network meta-analysis

**DOI:** 10.1097/MD.0000000000021496

**Published:** 2020-07-31

**Authors:** Yang Wang, Hui Li, Wei Peng, Yalin Chen, Mimi Qiu, Jun Wang, Qinghong Hao, Yang Tu, Yunlu Liu, Tianmin Zhu

**Affiliations:** a School of Rehabilitation and Health Preservation, Chengdu University of Traditional Chinese Medicine; b Department of Rehabilitation, Shuangliu Maternal and Child Health Care Hospital; c School of Medicine, Chengdu University; d School of Acupuncture and Tuina, Chengdu University of Traditional Chinese Medicine; e Institute of Laboratory Animal Sciences, Sichuan Academy of Medical Sciences & Sichuan Provincial People's Hospital; f Department of Pharmacy, Sichuan Academy of Medical Sciences & Sichuan Provincial People's Hospital, Chengdu, China.

**Keywords:** network meta-analysis, non-pharmacological interventions, postpartum depression, protocol

## Abstract

**Background:**

Postpartum depression (PPD) is one of the most common mental disorders in women following childbirth with heightened prevalence across the globe. Both pharmacotherapy and non-pharmacological interventions are effective for PPD. However, due to the concerns about the side effect on the mother and child of pharmacological treatments, most of women with PPD choose non-pharmacological therapies as their first line option. Prescription of these non-drug approaches should be guided by high quality evidence. Therefore, this network meta-analysis aims to compare, rank and interpret existed non-pharmacological evidence for the effective treatment of women with PPD.

**Methods:**

Electronic bibliographic databases including EMBASE, PubMed, Scopus, The Cochrane Library, Web of Science, China National Knowledge Infrastructure (CNKI),VIP Database, Wanfang Database and Chinese Biomedical Literature Database will be searched for relevant randomized controlled trials (RCTs) of non-pharmacological interventions for PPD. Heterogeneity and inconsistencies will be analyzed by I^2^ statistic and Z test, respectively. We will assess the quality of evidence by the Grading of Recommendations Assessment, Development and Evaluation (GRADE) and evaluate the risk of bias according to Cochrane risk of bias tool. R software 3.6.1 (R Foundation for Statistical Computing, Vienna, Austria) will be used to conduct a network meta-analysis.

**Results:**

Formal ethical approval is not required, because the present study is a meta-analysis based on existed studies. The findings of this research will be reported in a recognized journal.

**Conclusion:**

The review results will ascertain the hierarchy of effectiveness of different non-pharmacological approaches for PPD, and systematically provide suggests for physicians and patients.

**Trial registration number:**

PROSPERO CRD42020166801

## 1 Introduction

Postpartum depression(PPD) is the most common mental disorder for women following childbirth, which can deleteriously affect maternal behaviors and impair the cognitive, behavioral, emotional development of children.^[1]^ Epidemiological studies have found that women with PPD were at high risk for suicidality,^[2]^ and almost 1/3 women still reported depressive symptoms even 4 years after childbirth.^[3]^ Besides, the global pooled incidence of PPD is estimated to be 17.7%, which is up to 38% in Chile.^[4]^ As a consequence, PPD has been considered as a global public health issue.

Given the serious consequences of PPD for both mother and child and the high prevalence of PPD, effective treatment for this disease is of obvious importance. Drug therapy is the main treatment for major depressive disorder (MDD) in clinic and used cautiously during perinatal period.^[5,6]^ Due to concern about infant exposure to antidepressant via breastmilk, many postpartum women are reluctant to start antidepressant therapies.^[7]^ Moreover, PPD are often mixed with bipolar feature, which is usually hard to recognize, therefore inappropriate antidepressant therapies may lead to a deterioration of hypomanic symptoms and increase the risk of suicidality in mother.^[8]^ As a consequence, non-pharmacological interventions have become many PPD patients’ preferences.

Methods of psychological/ psychosocial intervention, physical therapy, kinesitherapy, music therapy, acupuncture are the widely used non-drug approaches for PPD,^[9]^ which have none of negative effects of pharmacotherapy mentioned above, therefore, women with PPD could continue breastfeeding. Cognitive behavioral therapy (CBT) and interpersonal psychotherapy (IPT) are 2 of the most common psychological/ psychosocial interventions with strong evidences on clinical efficacy for treating PPD.^[10,11]^ Physical therapies were also proved effective for PPD, such as repetitive transcranial magnetic stimulation (rTMS) and light therapy. Repetitive transcranial magnetic stimulation, which was widely used in MDD, can significantly improve the PPD symptoms.^[12]^ Furthermore, the efficacy of rTMS has been proved to last even for more than 6 months.^[3]^ Light therapy is another effective physical treatment method, targeting disordered sleep and circadian rhythms, which are main risk factors of PPD.^[13]^ Kinesitherapies, like aerobic exercise and yoga, are considered as the low-cost, freely available interventions for postpartum women with depressive symptoms, which have the potential to become more generally in PPD population.^[14,15]^ Music therapy, demonstrated effective to MDD patients, was also found curative for PDD.^[16]^ Additionally, as one of the most popular alternative medicines in the world, acupuncture therapy could alleviate the depressive and anxious symptoms of PPD women, as well as the estradiol levels.^[17]^

It can be clearly seen that there is a wide choice of non-pharmacological approaches for physicians, who need high quality evidence to back them to make the best decision in clinical practice. Therefore, to meet the increasing need for comparing the values of these non-drug interventions, a network meta-analysis should be conducted to fill in the gap for assessment of comparative efficacy and understand the relative merits of the various approaches, in the condition where the evidence is indirect.^[18]^ The systematic review and network meta-analysis based on high quality randomized controlled trials (RCTs) aim to inform clinicians by comparing and ranking different non-pharmacological interventions for precise and safety treatment of women with PPD. Furthermore, by analyzing individual treatments or combinatorial usage, we hope the results of the study will provide a wider picture for summarizing and interpreting the existed evidence data.

## 2 Methods

### 2.1 Design and registration

**Figure 1:**
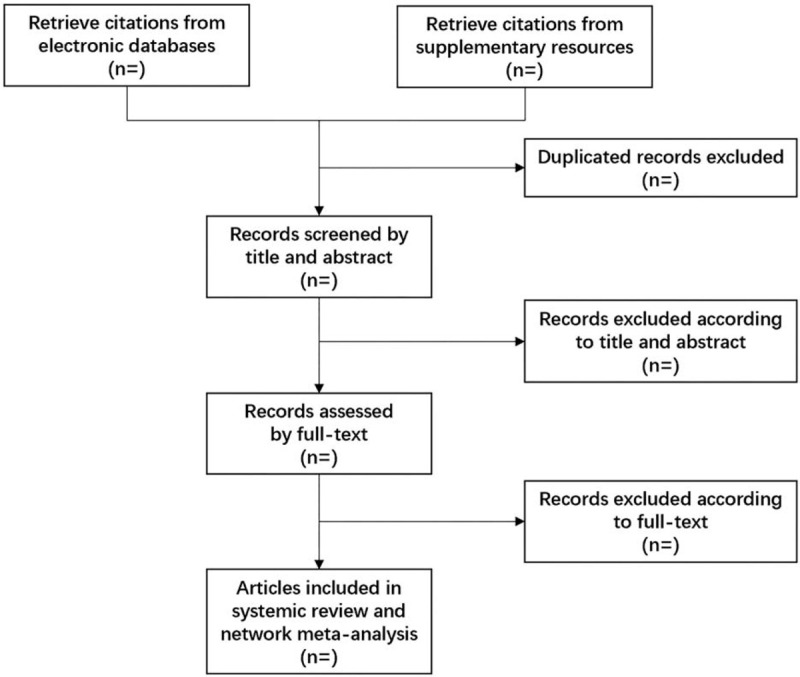
Flow chart of systematic review and network meta-analysis for non-pharmacological interventions for postpartum depression.

This systematic review and network meta-analysis will evaluate the comparative effectiveness and safety of non-pharmacological interventions for PPD. This protocol is designed according to the guideline of Preferred Reporting Items for Systematic Review and Meta-Analysis Protocols (PRISMA-P)^[19]^ (Fig. 1) and registered on the international prospective register of systematic review (PROSPERO), with the registration number CRD42020166801. The findings of this study will be reported in line with the guideline of Preferred Reporting Items for Systematic Reviews and Network Meta-Analysis (PRISMA-NMA).^[20]^

### 2.2 Ethics

Since the present study is a meta-analysis based on existed researches, formal ethical approval is not required.

### 2.3 Eligibility criteria

#### 2.3.1 Type of studies

All RCTs of non-pharmacological interventions for PPD will be included in this study, regardless of blinding. The written language is limited in English or Chinese.

#### 2.3.2 Type of participants

Participants were primiparous or multiparous postnatal women diagnosed with postpartum depression through a systematical clinical interview or a validated depression scoring system.

#### 2.3.3 Type of interventions and comparators

We will include the RCTs evaluating the effectiveness of non-pharmacological treatments in treating PPD. Psychological/psychosocial interventions (including cognitive behavioral therapy (CBT) and IPT), physical therapies (including rTMS and light therapy), kinesitherapies (including aerobic exercise and yoga), music therapy, and acupuncture (including traditional acupuncture, or electro-acupuncture, or auricular acupuncture) will be involved in our study.

There are no limitations on the administration methods and duration of treatments. Studies combining non-pharmacological treatments and pharmacological treatments will be excluded, while studies with concomitant use of different non-pharmacological interventions will be included.

The control group will include usual care, waiting-list control, non-treatment control, and placebo, which will be regarded as a single node in network meta-analysis.

#### 2.3.4 Outcome measures

The main outcome is the change in the depression score from baseline to endpoint, measured by validated depression scoring systems, such as Geriatric Depression Scale (GDS), Hamilton Depression Rating Scale (HAMD), Beck Depression Inventory (BDI), Self-Rating Depression Scale (SDS), Center for Epidemiologic Studies Depression Scale (CES-D), Edinburg Postpartum Depression Scale (EPDS), and Patient Health Questionnaire-9 (PHQ-9).

In order to examine the possible maintenance effects of non-pharmacological treatments, effect at the end of follow-up will also be evaluated as the secondary outcome.

#### 2.3.5 Exclusion criteria

We will exclude the researches with quasi-random allocation or the studies without available data. Duplicate publication will also be excluded in the current study.

### 2.4 Search strategy

A comprehensive search of the following electronic bibliographic database resources will be attempted for the identification of English data:

- EMBASE
- PubMed
- Scopus
- The Cochrane Library
- Web of Science

The following Chinese databases will also be searched for the identification of Chinese data

- China National Knowledge Infrastructure (CNKI)
- VIP Database
- Wanfang Database
- Chinese Biomedical Literature Database

Both English and Chinese databases were searched from their inception to March 15, 2020. In addition, we will also search the potential studies in reference lists of eligible studies, and the ongoing eligible trials in international trial registry websites.

The preliminary search strategy of PubMed is shown in Table 1, which will be adjusted in accordance with specific database.

**Table 1:**
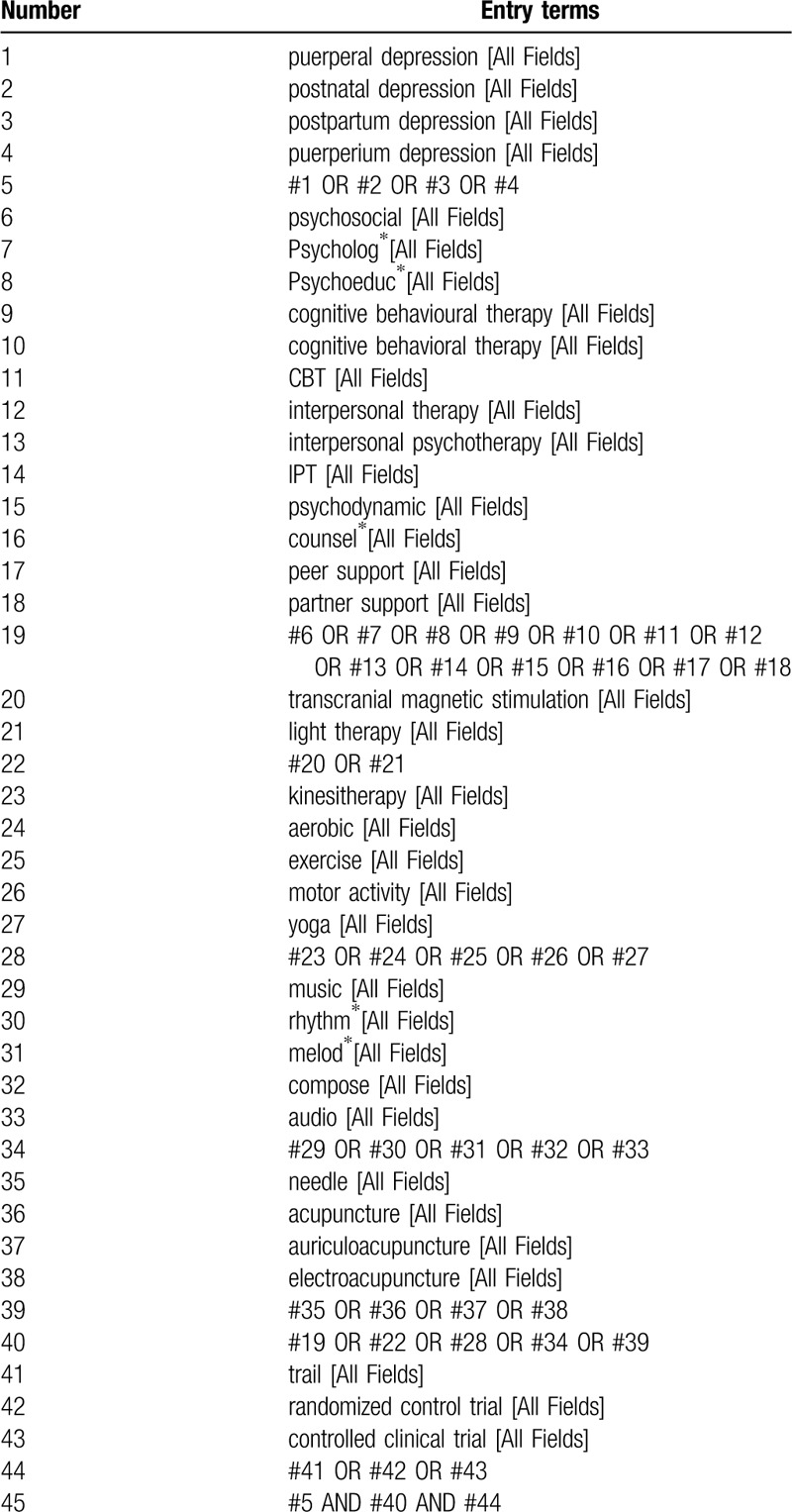
PubMed search strategy draft.

### 2.5 Study selection and data extraction

All databases mentioned above will be scanned using the pre-designed search strategy, and all retrieved articles will be imported into Endnote X7 (Thomson Reuters LLC, Philadelphia, USA). The duplicate reports will be deleted initially. Then 2 independent reviewers (YW and HL) will review the titles and abstracts of studies to select available RCTs in line with the eligibility criteria. Afterward, the full-text will be downloaded for further assessment. Finally, 2 reviewers (YW and HL) will independently extract data using a standardized extraction form, which includes:

(1) general information (e.g., authors, journal, year of publication, country, methods of randomization and blinding);
(2) participants (e.g., number, age, and severity of postpartum depression);
(3) interventions and controls (e.g., type of therapy, follow up period and control group);
(4) outcomes (the primary and additional outcomes).

Any disagreements will be resolved through discussion or consultation with a third reviewer (WP). If there is any missing data, we will try to contact the original author by email or telephone to obtain the complete data.

### 2.6 Assessment of risk of bias

Two independent reviewers (YC and MQ) will assess the risk of bias of included studies according to the Cochrane Handbook,^[21]^ which includes random sequence generation, allocation concealment, blinding of participants, personnel and outcome assessors, incomplete outcome data, selective reporting, and other sources of bias. We will classify each domain into 3 levels: high, unclear or low risk of bias. Any variation in opinion between reviewers will be resolved by discussion or consultation with a third reviewer (WP).

### 2.7 Data synthesis and statistical methods

#### 2.7.1 Pairwise and network meta-analysis

We will conduct the conventional pairwise meta-analysis for direct comparisons. The effect size of continuous variable data will be calculated with standardized mean difference (SMD), while the effect size of categorical variable data with risk ratio (RR). The related 95% confidence intervals (CIs) of SMD and RR will also be computed.

For indirect comparisons, a network meta-analysis is required to mix results to improve statistical efficacy. The network meta-analysis will be performed with the “netmeta”package of R software (R Foundation for Statistical Computing, Vienna, Austria), which based on frequentist method.^[22]^ The direct and indirect comparison results will be presented as a network diagram.

#### 2.7.2 Assessment of heterogeneity

Before the combination of effect size, the homogeneity will be analyzed by I^2^ statistic to check whether the results of individual studies are mergeable. When I^2^ ≤ 50%, the fixed effect model will be used to combine the effect size. Conversely, the random effect model will be used for meta-analysis after excluding the influence of apparent clinical heterogeneity.

#### 2.7.3 Subgroup and sensitivity analysis

The subgroup analysis will be conducted to resolve the potential high heterogeneity across RCTs.

We will conduct sensitivity analysis to verify the robustness of the results. The research with unclear or high risk of bias will be exclude to check if the results would change.

#### 2.7.4 Assessment of inconsistency

Consistency is a basic principle in network meta-analysis, which indicates the consistency between direct comparative result and indirect comparative results. As a loop is established among interventions, inconsistency will be evaluated by Z test.^[23]^ The Z-value and its corresponding *P*-value will be calculated, and the inconsistencies exist between the different comparisons if the *P*-value < .05.

#### 2.7.5 Publication bias

We will also evaluate potential publication bias using funnel plots and Egger test.^[24]^

### 2.8 Grading the quality of evidence

The evidence quality will be evaluated by 2 independent reviewers (JW and QH) using the Grading of Recommendations Assessment, Development and Evaluation (GRADE),^[25]^ which classifies the quality of evidence as high, medium, low, and very low.

## 3 Discussion

The designation of this network meta-analysis protocol referred to the previous work.^[5,26]^ Accumulating studies have provided options of using non-pharmacological interventions in PPD clinical practice. However, the best decision of non-pharmacological approaches is hard to make for physicians to treat woman with PPD, due to the lack of strong evidence base. The current clinical studies of verifying the efficacy of different non-pharmacological methods are rarely compared between each other directly. The network meta-analysis provides us a way to compare, rank, interpret the indirect evidence base of multiple non-drug therapies. We hope the results of the present study will help the clinicians to prescript a suitable single or mixed non-pharmacological treatment for women with PPD.^[27]^

## Author contributions

**Conceptualization:** Yang Wang, Yunlu Liu, Tianmin Zhu.

**Data curation:** Yang Wang, Hui Li.

**Formal analysis:** Yalin Chen, Mimi Qiu.

**Funding acquisition:** Tianmin Zhu, Yunlu Liu.

**Investigation:** Yang Wang, Hui Li, Yalin Chen, Mimi Qiu, Jun Wang, Qinghong Hao, Yang Tu.

**Methodology:** Yang Wang, Wei Peng.

**Supervision:** Tianmin Zhu.

**Writing – original draft:** Yang Wang, Yunlu Liu.

**Writing – review & editing:** Yunlu Liu, Tianmin Zhu.
